# Mechanically Reconfigurable Single-Arm Spiral Antenna Array for Generation of Broadband Circularly Polarized Orbital Angular Momentum Vortex Waves

**DOI:** 10.1038/s41598-018-23415-1

**Published:** 2018-03-23

**Authors:** Long Li, Xiaoxiao Zhou

**Affiliations:** Key Laboratory of High Speed Circuit Design and EMC of Ministry of Education, School of Electronic Engineering, Collaborative Innovation Center of Information Sensing and Understanding, Xidian University, Xi’an, 710071 China

## Abstract

In this paper, a mechanically reconfigurable circular array with single-arm spiral antennas (SASAs) is designed, fabricated, and experimentally demonstrated to generate broadband circularly polarized orbital angular momentum (OAM) vortex waves in radio frequency domain. With the symmetrical and broadband properties of single-arm spiral antennas, the vortex waves with different OAM modes can be mechanically reconfigurable generated in a wide band from 3.4 GHz to 4.7 GHz. The prototype of the circular array is proposed, conducted, and fabricated to validate the theoretical analysis. The simulated and experimental results verify that different OAM modes can be effectively generated by rotating the spiral arms of single-arm spiral antennas with corresponding degrees, which greatly simplify the feeding network. The proposed method paves a reconfigurable way to generate multiple OAM vortex waves with spin angular momentum (SAM) in radio and microwave satellite communication applications.

## Introduction

Recently, with modern communication systems developing rapidly, flourishing reformations are needed for radio transmissions^1^. The concept of orbital angular momentum (OAM) has attracted tremendous interest owing to its distinctive property of transmitting multiple signals simultaneously at the same frequency, which may be used to potentially improve spectrum efficiency^2–4^. In 2007, Thidé *et al*.^5^ theoretically utilized antenna arrays to generate OAM beams whose characteristic is similar to the Laguerre-Gaussian laser beams in the low-frequency radio domain. Then, the vortex beams generated by circular antenna arrays has been proposed by Mohammadi *et al*.^6^. Later, the experiment test on the OAM vortex wave was carried out by Tamburini *et al*.^7^, which reveals that OAM radio beams can be applied to enhance the wireless spectral efficiency. Since then, the application of OAM in radio frequency domain has attracted widespread attention.

For OAM generations, various efficient approaches have been proposed. Spiral phase plates (SPPs) have been fabricated for the twist radio beams, which transform a Hermite-Gaussian mode into a Laguerre-Gaussian mode, and then improved SPPs of various types are widely used in optics as they allow structures simple and easy to implement^8–13^. However, the dielectric loss and reflection of SPPs will decrease the performance, which limit the application in low-frequency radio domain. Meanwhile, a lot of efficient parabolic, spiral reflector antennas are designed for OAM transformation. Generally, the circular array antennas are widely predicted to generate vortex beams carrying OAM in radio frequency domain. On the other hand, the circular array antennas that can be utilized to form OAM beams of different mode numbers are studied^14^, which create multiple sub-channels of propagation corresponding to the twisting degree of the electromagnetic wave and enable OAM modes multiplexing with good channel isolations^15^. However, a complex feeding system should be implemented in order to obtain a rotating phase fronts radio beam. Hereafter, some metasurfaces were proposed to generate single, multiple, dual- polarization and dual-mode orbital angular momentum, etc.^15–18^.

In this paper, we focus on the design, fabrication and experimentally demonstrations of the mechanically reconfigurable circular array antennas which can produce various OAM modes with circular polarization in broad bandwidth. Nowadays, numerous applications of linear polarization on the OAM mode have attracted widespread attention, while other researches and experiments are rarely related to the circular polarization which can avoid the polarization mismatch effectively in the space communications and double the spectrum capacity and channel quantity.

## Results

### Design method of circular antenna array

**Figure 1:**
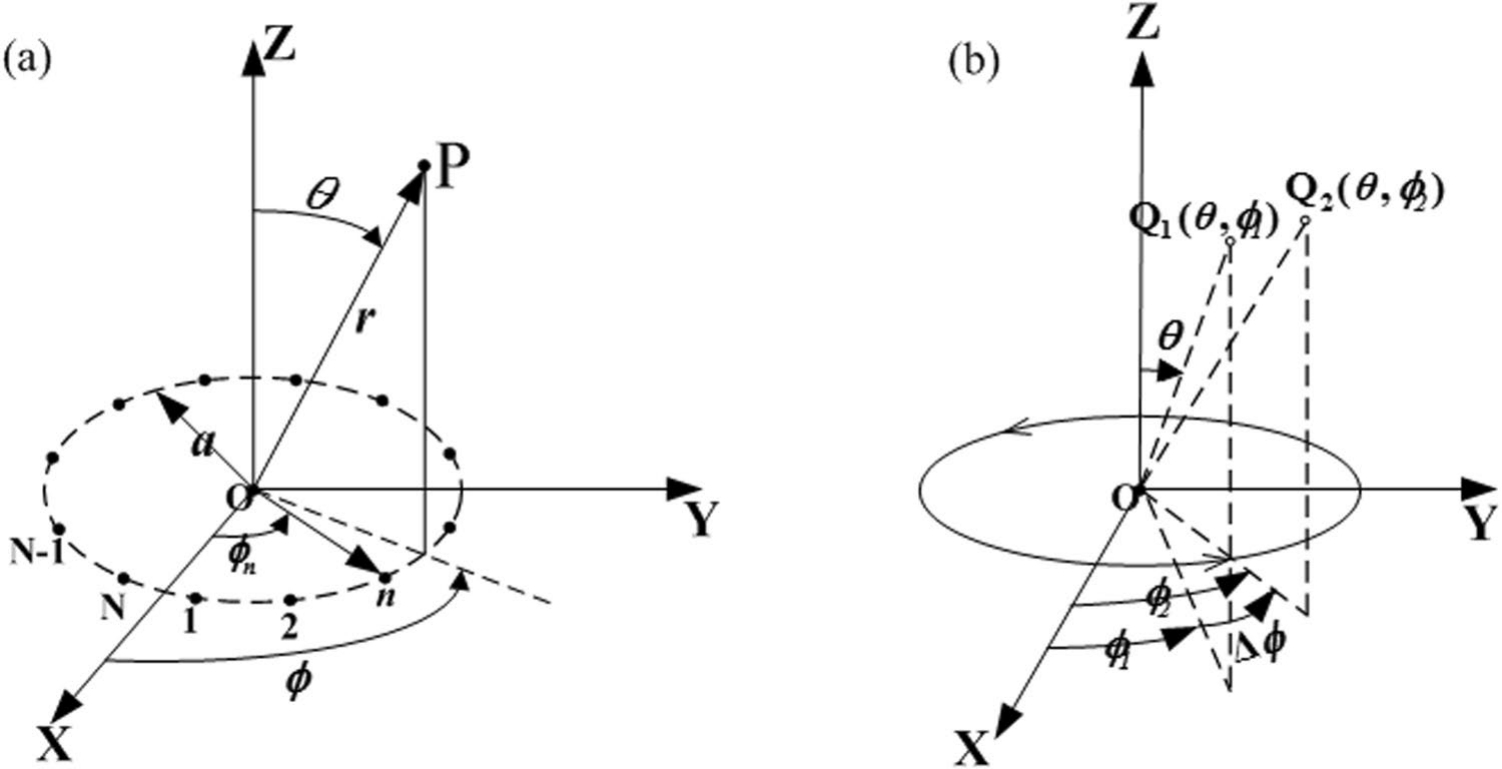
(**a**) Schematic diagram. (**b**) Phase properties of a uniform circular current distribution.

A schematic diagram of the circular antenna array in the free space is illustrated in Fig. 1(a). The upper point *P* is the receiving point in the far zone, and the lower points are the elements of the transmitting array which will be used to generate OAM beams. *N* isotropic array elements are equally spaced on the *x*-*y* plane along a circular ring with the radius *a*, the distance between the point P in the far field and the center point O of the circular ring is *r*, as shown in Fig. 1(a).

The excitation source of the *n*th array element is assumed as

1 $${{I}}_{{n}}={I}{{e}}^{-{j}{{\beta }}_{{n}}}\quad (n=1,2,3,\ldots ,N)$$

where *I* is the amplitude, *β*_*n*_ is the phase of the *n*th excitation signal and *N* is the number of the elements of the array. According to the electromagnetic far field principle of superposition, the electric field (*E*) in the far-field zone can be expressed as

2 $$E=\frac{{e}^{-jkr}}{r}\sum _{n=1}^{N}I{e}^{-j[ka\sin \theta \cos (\varphi -{\varphi }_{n})+{{\beta }}_{n}]}$$

where *ϕ* is the phase of the element below the receiving point and *ϕ*_*n*_ is the phase of the *n*th element, which is expressed as

3 $${\varphi }_{n}={\varphi }_{0}+n\cdot 2\pi l/N$$

where *ϕ*_0_ is the reference phase. From the Eq. (2), the array factor *f*_*a*_ can be expressed as

4 $${f}_{a}=\sum _{n=1}^{N}I{e}^{-j[ka\sin \theta \cos (\varphi -{\varphi }_{n})+{\beta }_{n}]}$$

As mentioned above, when *N* is infinitely close to ∞, the Eq. (4) can be assumed as

5 $${f}_{a}={K}_{1}{e}^{-jH\varphi }{J}_{H}(ka\,\sin \,\theta )$$

where *K*_1_ = *N* and *H* = *β*_*n*_/*ϕ*_*n*_. The two constants *K*_1_ and *H* are respectively dependent on the reference antenna and antenna current relationship. It is assumed that the phase of the current satisfies the following relation

6 $${\beta }_{n}={l}\cdot {n}{\rm{\Delta }}\varphi $$

where *l* is the mode of the OAM^19,20^, hence we have

7 $$H=l\cdot n{\rm{\Delta }}\varphi /n{\rm{\Delta }}\varphi =l$$

From above, the phase factor *e*^−*jHϕ*^ would be translated into *e*^−*jlϕ*^, which illustrates that the orbital angular momentum vortex beam can be generated effectively by controlling the excitation phase of the array elements. The largest OAM mode number which the *N*-element array can generate is −*N*/2 < *l* < *N*/2^5^.

### Design of single-arm spiral antenna

The circular antenna array employed in this paper consists of single-arm spiral antennas (SASAs) which allow self-rotation around the axis to produce a relative change in phase of the radiated field in space. One degree of mechanical rotation produces a corresponding change in phase of one electrical degree. For simplicity, we assume the spiral antenna array as a circular ring conductor firstly, which supports a uniform progressive current wave. As shown in Fig. 1(b), the arrows on the circular conductor indicate the direction in which current phase fronts move. Consider two points in the far field of this circular current distribution, *Q*_1_(*θ*, *ϕ*_1_) and *Q*_2_(*θ*, *ϕ*_2_), whose spherical coordinates differ only in the azimuth coordinate *ϕ*. It is clear that these two points see the same current distribution except for a shift of phase in the current sources along the circle, that is the sources for the radiation field at *Q*_2_ are identical with the sources for the radiation field at *Q*_1_ except for a phase lag in the sources for *Q*_2_ over those for *Q*_1_, of Δ*ϕ* electrical degrees. Accordingly, the radiation field of the circular current loop depends only with respect to phase on the azimuth coordinate *ϕ* this dependence is given by the factor *e*^−*jϕ*^. It follows immediately that a rotation of the circular current loop, which does not disturb the intrinsic current phasing, changes the phase of rotation field of loop everywhere by an amount which in electrical degrees is precisely equal to the number of degrees of mechanical rotation^21^. Phase properties of the single-arm spiral antenna are different from the Pancharatnam-Berry structure which can produce phase delay by changing the direction of the incident electric field^22,23^.

**Figure 2:**
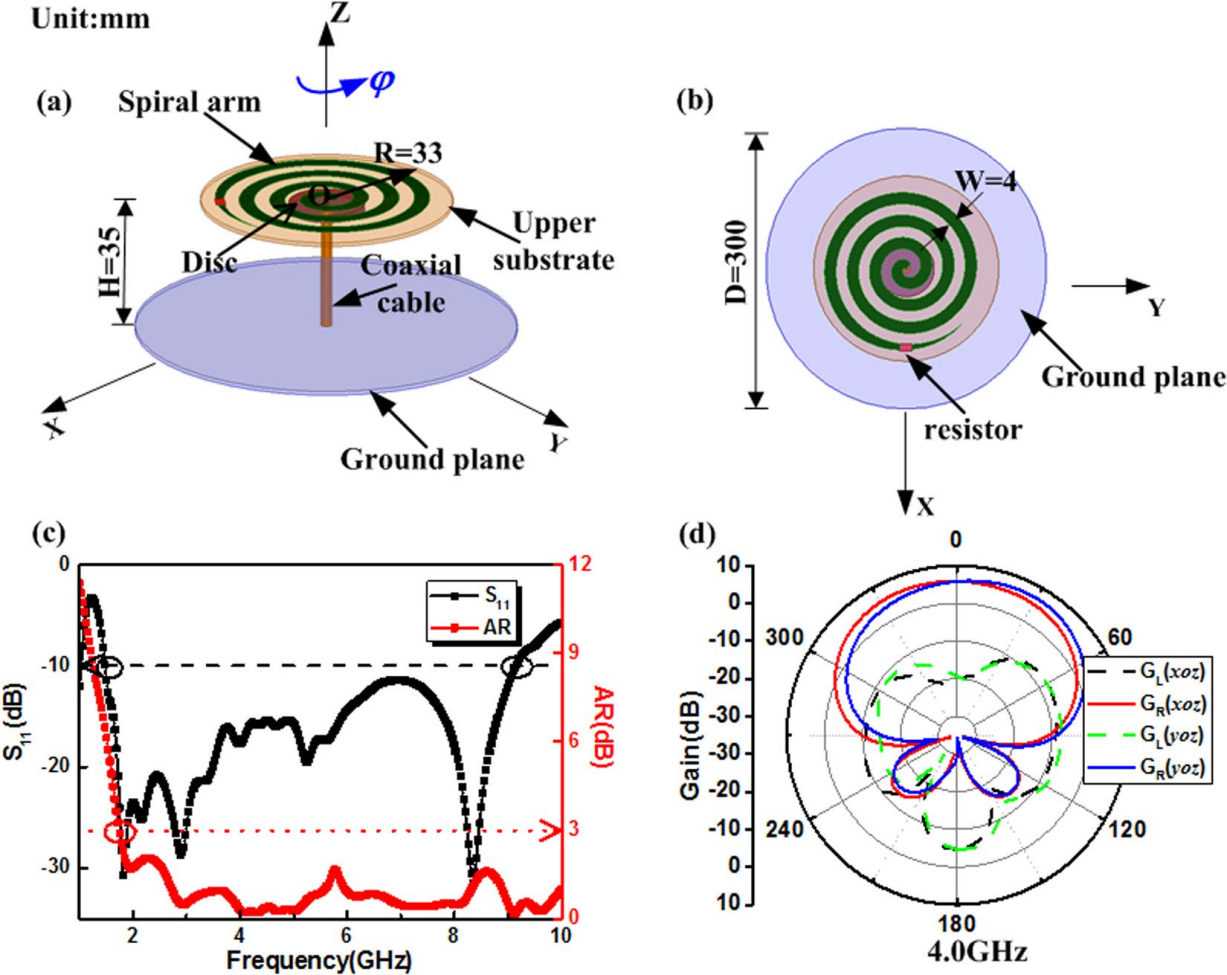
Geometry and simulated results of the single-arm spiral antenna. (**a**) Solid view of the single-arm spiral antenna. (**b**) Top view of the single-arm spiral antenna. (**c**) S_11_ and axial ratio characteristics of the designed single-arm spiral antenna. (**d**) Radiation patterns of the single-arm spiral antenna in x-z plane and y-z plane at 4.0 GHz.

As shown in Fig. 2(a) and (b), a single-arm spiral antenna can be designed to generate the right-hand (RH) circular polarization electromagnetic wave^24^. The antenna arm made of a conducting spiral strip at the height H from the ground plane, which is printed on a 1-mm-thick dielectric substrate with relative permittivity 2.65 and dielectric loss tangent 0.003. Specifically, a 160 Ω termination resistor is used to slowly attenuate the current reflection from the spiral end. The rotation phase of the printing spiral arm along the z-axis is defined as *φ*. And there is a lower substrate of same property with 2 mm thickness under the upper one. The disc, which is behind the spiral and printed on the bottom of the lower substrate, has a function of impedance matching. The single-arm spiral antenna is fed by using a 50 Ω coaxial cable, where the inner and outer conductors of coaxial cable are connected to the spiral and the disc, respectively. A metallic ground plane has a radius of 50 mm, which is used as the reflector of the spiral antenna.

Figure 2(c) shows the simulated characteristics of the proposed SASA. It can be seen that the S_11_ is less than −10 dB over the broadband ranging from 1.5 to 9 GHz, and the axial ratio (AR) is less than 3 dB ranging from 1.73 to 9 GHz. Figure 2(d) shows the radiation patterns in *x*-*z* plane and *y*-*z* plane at 4 GHz. The radiation pattern is illustrated with a right-hand CP component G_R_ and a left-hand CP component G_L_. It is found that the radiation pattern of G_R_ is larger than the G_L_ at least 20 dB, which realizes the degree of the cross polarization of SASA is low in the normal direction. In a word, the proposed single-arm antenna is a broadband circularly polarized antenna which can generate a change in phase of the radiated field by mechanically rotating the spiral arm.

### Design of circular antenna array with eight single-arm spiral antennas

It is an effective way to generate OAM-carrying radio beams using a circular antenna array. In this paper, the circular array consists of eight identical SASAs whose excitations have the same magnitude and phase, but the phase difference required for different OAM modes can be achieved by mechanically rotation of the SASA. In this case, the OAM mode numbers with *l* = 0, ±1, ±2, ±3 can be generated effectively.

**Figure 3:**
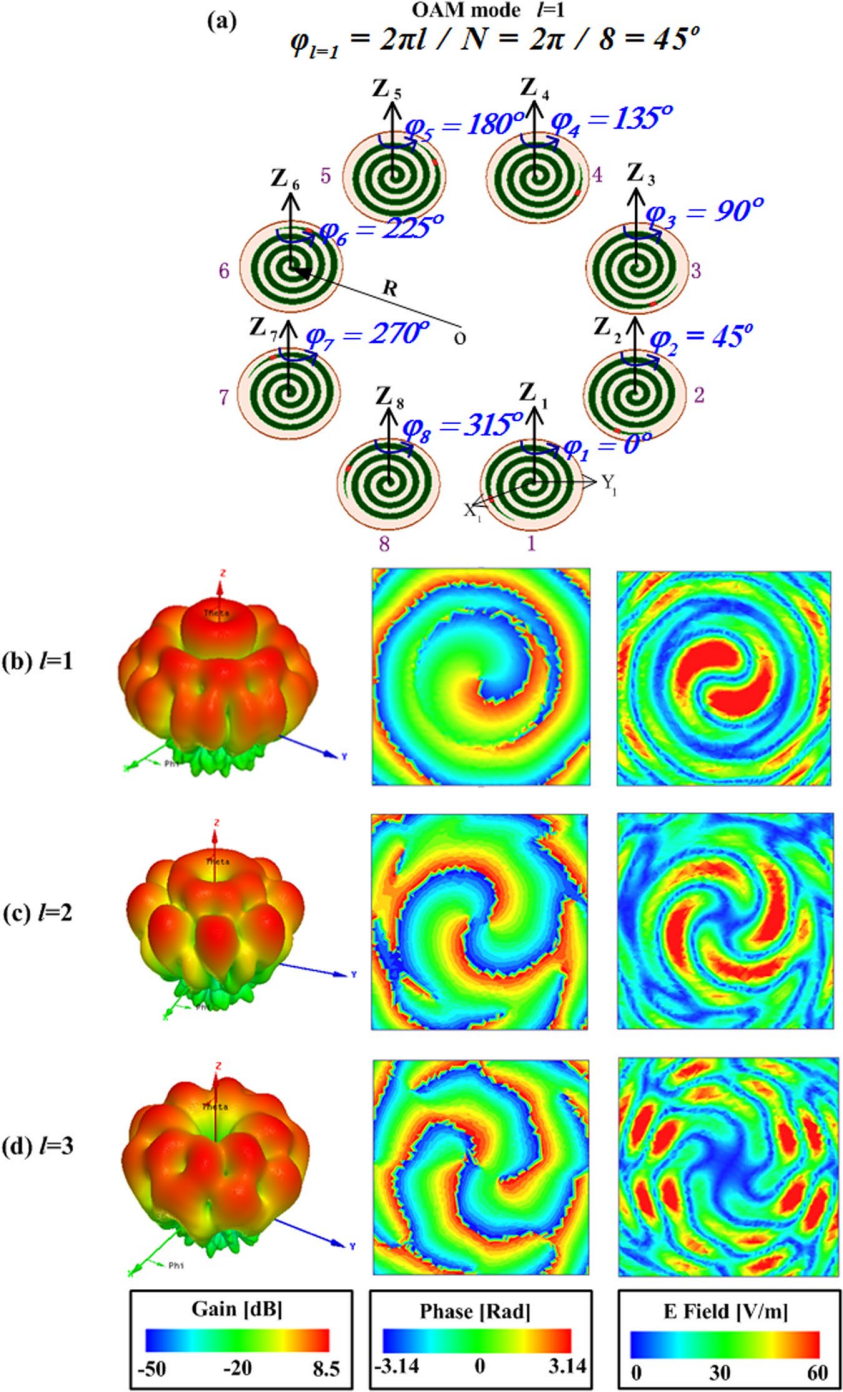
(**a**) Geometry of circular antenna array with SASAs for generation of vortex wave with OAM mode 1. Far-zone *E*-field and near-zone *E*-field distribution of OAM beam with different modes of (**b**) *l* = 1, (**c**) *l* = 2, and (**d**) *l* = 3.

The magnitude of the surface current on the SASA is almost equal due to the property of the SASA that is circular symmetry about the axis. According to its rotation property, the correspondence of mechanical rotation produces a relative change in phase between two adjacent SASAs. So taking SASAs as elements to form array antennas, the first SASA served as the reference zero-phase and initial position, the other seven SASAs can be rotated clockwise or anticlockwise on their own axes with reference degree to generate negative or positive modes of the orbital angular momentum vortex waves. As shown in Table 1, the correspondence of OAM modes and rotation angles between two adjacent SASA elements are illustrated, and the positive phase-shift can be produced by anticlockwise rotation and the negative phase-shift can be produced by clockwise rotation. Figure 3(a) presents an array consisting of 8-element SASA along a circular ring with the radius R = 120 mm working with mode *l* = 1, which is chosen as the example to explicate the Table 1. The 1^st^ SASA is taken as the benchmark without any rotation, and the *n*^th^ SASA rotates (*n* − 1) × 45° along its own axis (z-axis), respectively, which guarantees the phase delay between two adjacent SASA elements is always 45° and the whole array to produce the vortex wave with OAM mode *l* = 1. The position of resistor is selected as the rotating point for reference in this design. In this section, the positive modes *l* = 1, 2, 3 are used as examples to verify the OAM characteristics by adjusting the rotation angle of the SASA elements.

**Table 1:** Correspondence of OAM modes and self-rotation angles between two adjacent SASA elements.

OAM Mode	Phase delay (*φ*_*l*_ _=_ _*N*_ = 2*πl*/*N*)	Phase shift between nearby elements	Rotation angles of the *n*th SASA (*φ*_*n*_, n = 1, 2, ..., 8)
*l* = −3	−135°	*φ*_*n*_ − *φ*_*n* − 1_ = −135°	−(*n*−1) × 135°
*l* = −2	−90°	*φ*_*n*_ − *φ*_*n* − 1_ = −90°	−(*n*−1) × 90°
*l* = −1	−45°	*φ*_*n*_ − *φ*_*n* − 1_ = −45°	−(*n* − 1) × 45°
*l* = 0	0°	*φ*_*n*_ − *φ*_*n* − 1_ = 0°	0°
*l* = 1	45°	*φ*_*n*_ − *φ*_*n* − 1_ = 45°	(*n*−1) × 45°
*l* = 2	90°	*φ*_*n*_ − *φ*_*n* − 1_ = 90°	(*n* − 1) × 90°
*l* = 3	135°	*φ*_*n*_ − *φ*_*n* − 1_ = 135°	(*n* − 1) × 135°

Figure 3(b–d) give the simulated radiation pattern in the far-field zone at 3.5 GHz, an amplitude null can be seen in the center of the beam, which is the typical characteristic for OAM beams. In order to observe another properties of the vortex waves, the E-field intensity and phase on the near-field plane are shown in Fig. 3, which illustrates vortex beams can be successfully generated by using the mechanical reconfiguration property of the SASA element.

### Design of feeding network

**Figure 4:**
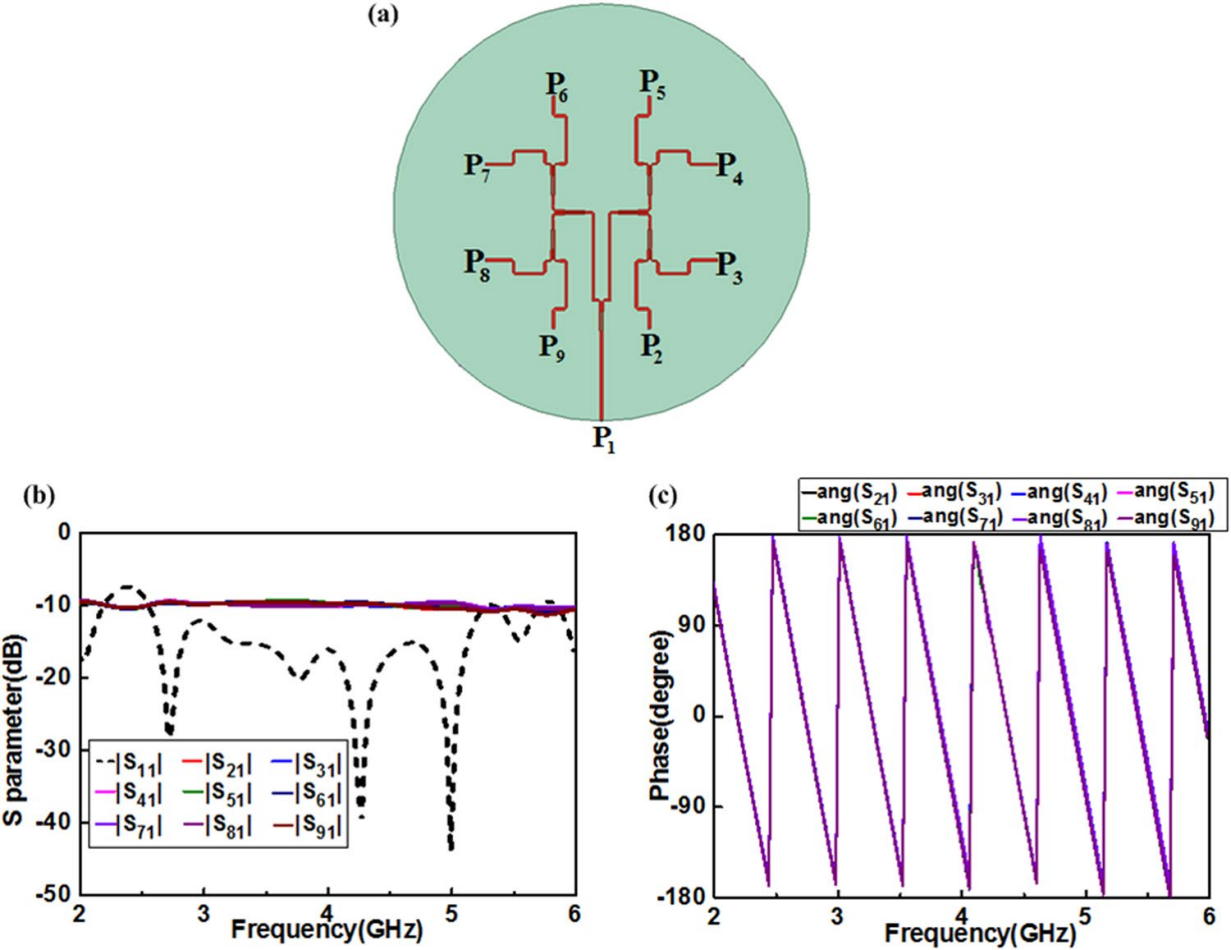
Geometry and simulated results of the feeding network. (**a**) Top view of the feeding network, (**b**) S-parameters characteristics of the feeding network, and (**c**) phases characteristics of the feeding network.

In this work, the feed network can be simplified as a uniform power divider whose amplitude and phase of eight output ports (P_2_ to P_9_) are all same to different OAM modes due to the rotation property of SASAs, which can bring a required change in phase only by rotating the single-arm spiral antenna with the corresponding phase. As shown in Fig. 4(a), the feeding network contains one input port (P_1_) and eight output ports (P_2_ to P_9_). The simulation results of the designed feeding network are presented in Fig. 4(b) and (c). The reflection coefficient of the input port is less than −10 dB and the transmission coefficients are between −9.4 dB and −10 dB with the magnitude imbalance smaller than 1 dB at 2.5–5.3 GHz. The phases of signals are all equal between each neighbor two output ports during 2.5–5.3 GHz. All the results have shown the excellent performances of the feeding network for precisely signals feeding during 2.5–5.3 GHz.

### Fabrication and measurement of the circular antenna array

**Figure 5:**
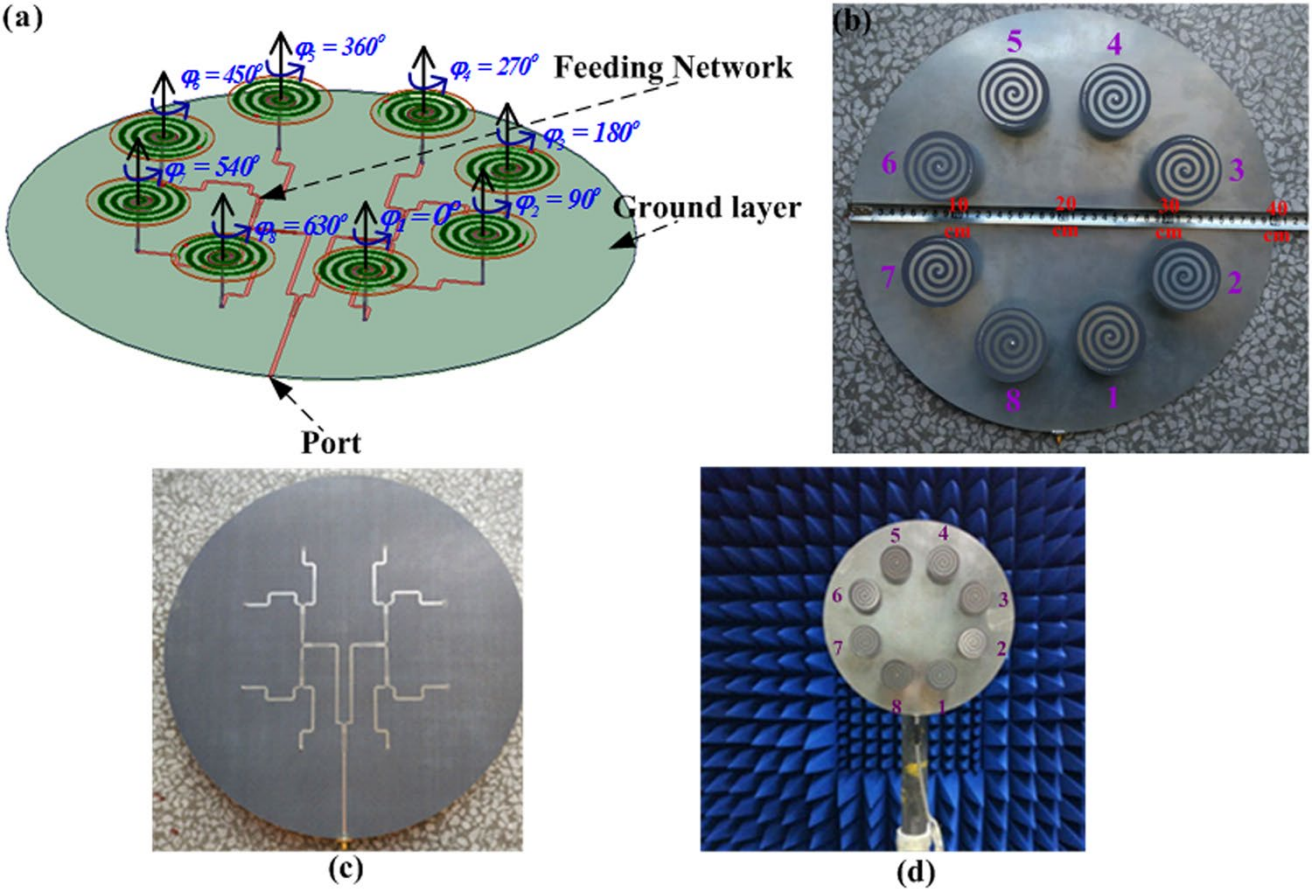
(**a**) Geometry configuration of the SASAs array, (**b**) top view of the fabricated OAM-generating SASAs array with OAM mode 2, (**c**) bottom view of the fabricated OAM-generating SASAs array feeding network, and (**d**) Experiment system configuration for far-field measurement.

As shown in Fig. 5(a), basically for each of the proposed SASAs, it is generally composed of eight elements and a ground layer with 1 mm thickness, the feeding network is at the bottom of the ground layer with inners connected from the spiral arms to the output ports of the feeding network and the ground plane is at the top of the ground layer with outers connected to the discs.

**Figure 6:**
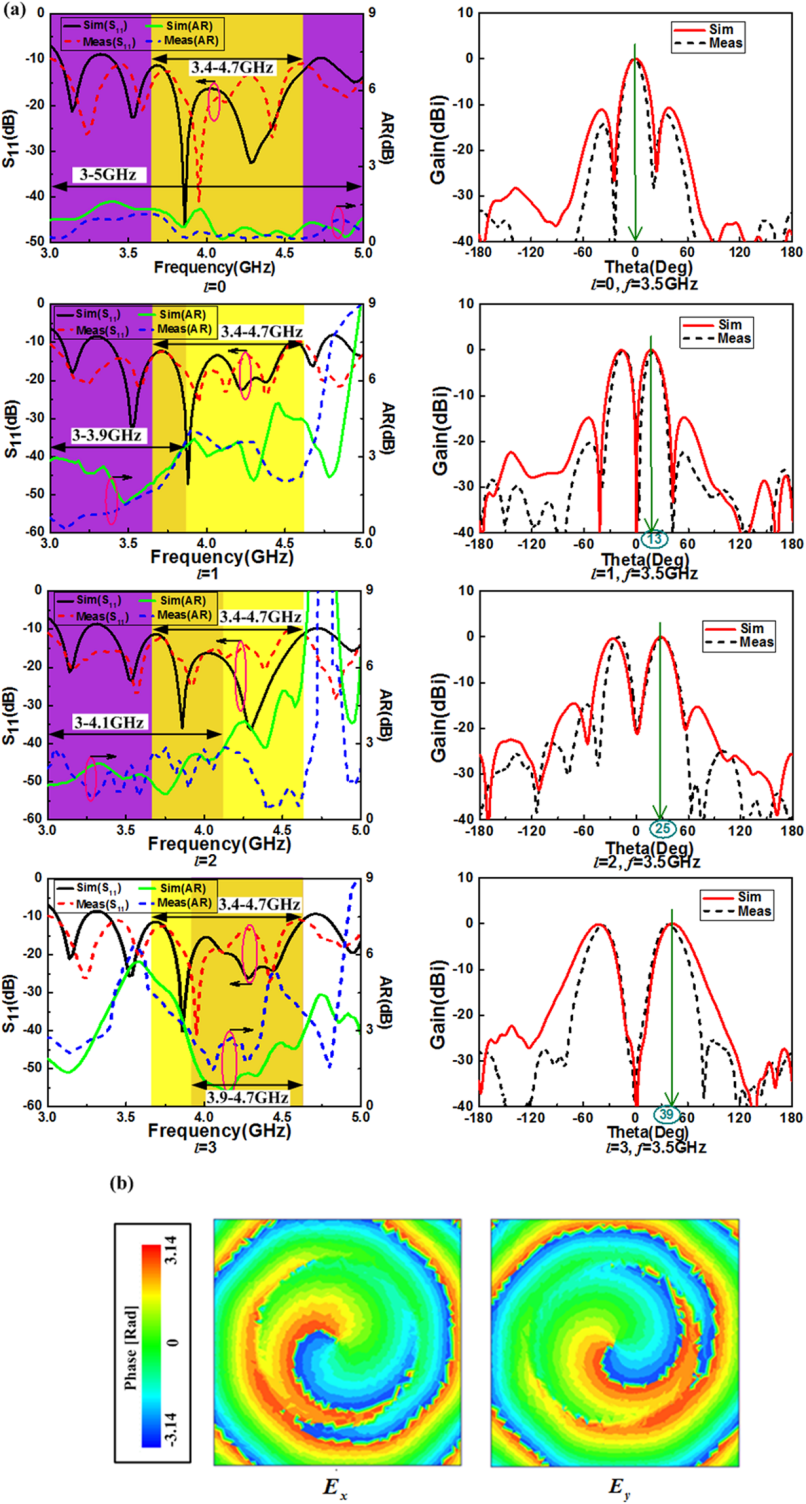
(**a**) Simulated and measured S-parameter, AR and 2D far-field radiation pattern of the circular array antenna with different OAM modes. Yellow and purple fill areas indicate the operate bandwidth of S_11_ and AR, respectively. (**b**) Simulated phase distributions of electrical field components E_*x*_ and E_*y*_ at 3.5 GHz.

In order to check the mechanical reconfiguration of the circular array, the measurement bandwidth is chosen from 2 GHz to 5 GHz because the power division network limits the frequency band, but it does not affect the inspection of mechanical reconfiguration. The prototype of OAM-generating SASAs array was fabricated and measured as shown in Fig. 5(b–d), the mode *l* = 2 is illustrated in the legend and other modes of OAM vortex beams are produced using mechanical reconfiguration property of the circular antenna array consisting of eight identical SASAs rotating reference degree as listed in Table 1. The measurements have been carried out with an Agilent network analyzer. The reflection coefficient |S_11_| is less than −10 dB at 3.4–4.7 GHz with OAM mode *l* = 0, 1, 2, 3 from the simulation results as shown in Fig. 6(a), which illustrates the operating band of SASAs array is broad and the measured results are consistent with the simulated results as a whole. Two aspects can be used to measure the performance of circular polarization. As for antenna array, the AR along the main lobe less than 3 dB is used to evaluate circular polarized property. From Fig. 6(a), the AR is less than 3 dB at 3.4–4.7 GHz with OAM mode *l* = 0.

For the AR bandwidth of OAM modes *l* = 1, 2, 3, respectively, it should be defined according to the main beam direction, in other words, we should consider the divergent angle of different OAM mode. Figure 6(a) shows that the AR bandwidth of OAM modes *l* = 1, 2, 3 are 3.4–3.9 GHz, 3.4–4.1 GHz, 3.9–4.7 GHz respectively. For OAM beams, the phase delay of the starting points of E_*x*_ and E_*y*_ phase distribution is taken to distinguish the circular polarization. The mode *l* = 1 is used as an example to verify the circular polarization, Fig. 6(b) shows phase delay of E_*x*_ and E_*y*_ phase distribution is 90 degree, which shows the polarization of the vortex beam is circular. In general, different OAM modes generated by circular antenna array with mechanical reconfiguration characteristic of SASAs have broadband and circular polarization.

**Figure 7:**
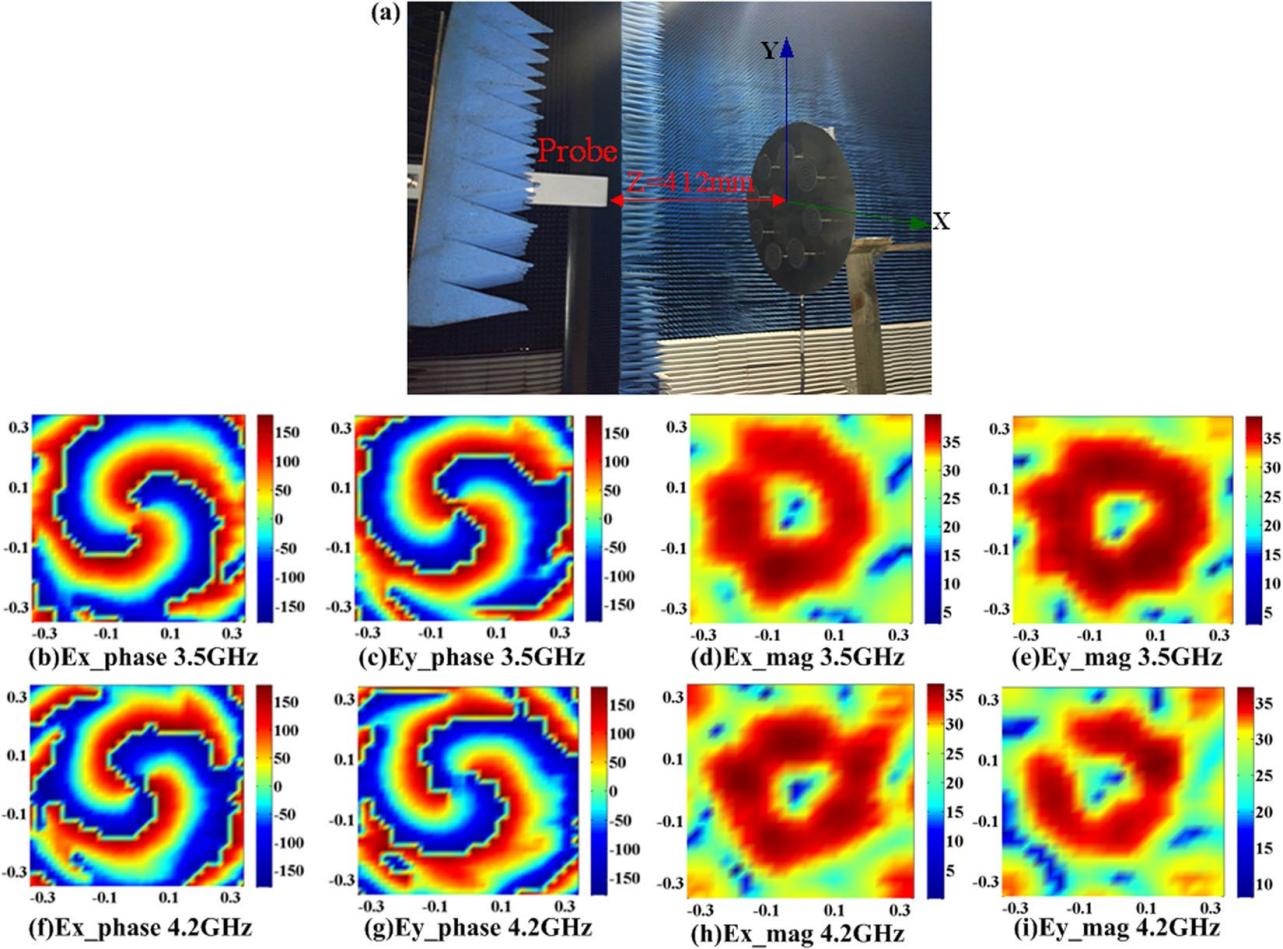
(**a**) Experimental system configuration for the OAM vortex wave measurement using near-field scanning technique. Measured electrical field characteristic with OAM mode *l* = 2 on the observational planes, (**b**) Ex-phase at 3.5 GHz, (**c**) Ey-phase at 3.5 GHz, (**d**) Ex-mag at 3.5 GHz, (**e**) Ey-mag at 3.5 GHz, (**f**) Ex-phase at 4.2 GHz, (**g**) Ey-phase at 4.2 GHz, (**h**) Ex-mag at 4.2 GHz, and (**i**) Ey-mag at 4.2 GHz.

For near-field sampling measurement, the example of mode *l* = 2 is used to verify characteristics of the OAM. Figure 7(a) shows the experimental scene of near-field sampling measurement. Figure 7(b–i) show the spatial phase distributions on the observational plane at different frequency points can be clearly recognized, the doughnut-shaped E-field intensity maps can also be observed. With comparison between (b) and (c), (f) and (g), the phase delay of 90° can be observed at the starting point of the rotating arms, respectively, which represents the OAM vortex wave generated by the proposed circular array is circularly polarized, which includes the OAM and SAM properties simultaneously.

## Methods

The numerical simulations of the reconfigurable single-arm spiral antenna element and array are implemented by HFSS based on the finite element method. The electric field distribution is obtained using the HFSS-IE domain boundary for a reduction of the simulation calculation. The far field measurements are performed by the free space method, where a horn antenna is connected with one port and the circular array with SASAs is connected with another port of the vector network analyzer to transmit and receive microwave signals. The circular array is placed at a distance of 2.8 m that meets the far-field requirement from the horn antenna. To measure the OAM vortex wave-front, the near-field planar scanning technique was performed between the operation frequency bandwidth from 2 GHz to 5 GHz. The vertical and horizontal polarization component of the electric field were detected by using standard measuring probes with three frequency bands to cover the entire measurement band. The experiment system is shown in Fig. 7(a), the near-field sampling plane is set at z = 412 mm with 20 mm sampling grid period, and the magnitude and phase of electrical field were measured on the sampling plane.

## Discussion

Using the array pattern described in Fig. 5(a), we experimentally realize mechanical reconfiguration with different rotation degrees corresponding to various OAM modes as shown in Table 1. Figure 6(a) depicts the simulated and measured results of far field of the circular array. As expected from the theoretical predictions, different modes of OAM vortex beams can be clearly observed. However, various OAM modes are achieved with mechanical reconfiguration of the circular array consisting of SASAs rather than using different feeding networks in this paper. As shown in Fig. 6(a), broadband and circularly polarized vortex beams can be achieved and the measured results show agreement with the simulation results, and slight discrepancy between the two results is associated with the fabrication tolerances and experimental errors.

In summary, our experiments show that circular array with single-arm spiral antennas can be used to effectively generate broadband and circularly polarized vortex beams, which can also be mechanically reconfigurable by rotating the spiral arms with corresponding degrees. This method greatly simplifies the feeding network from a complex power phase shifter to a simple power divider. As a result, the OAM vortex waves with different mode numbers can be conveniently realized. By using the proposed method, it is much easier to produce the vortex radio waves with different mode numbers comparing with traditional circular arrays, which provides a simple way to generate the OAM vortex waves for radio and microwave wireless communication applications.
